# Clinical lactation studies. Acting on key recommendations over the last decade

**DOI:** 10.1038/s44294-025-00064-0

**Published:** 2025-02-28

**Authors:** Karen Rowland Yeo, Jacqueline Gerhart, Aarti Sawant-Basak, Francis Williams Ojara, Aida N. Kawuma, Catriona Waitt

**Affiliations:** 1 Certara UK Limited (CPT Division), Sheffield, UK; 2 Pfizer Inc, Research and Development, 500 Arcola Road, Collegeville, PA 19424 USA; 3 AstraZeneca, 35 GateHouse Drive, Waltham, MA 02451 USA; 4 Infectious Diseases Institute, Makerere University College of Health Sciences, Kampala, Uganda; 5 Gulu University, Gulu, Uganda; 6 Department of Women’s and Children’s Health, University of Liverpool, Liverpool, UK

**Keywords:** Reproductive biology, Drug therapy

## Abstract

Including lactating women in clinical trials is imperative to generate relevant drug exposure and safety data needed to advise on clinical use of drugs in this understudied population. Recent changes in perspectives, regulatory guidance, and international networks which outline pragmatic approaches for advancing the conduct of clinical lactation studies are discussed. Case studies demonstrating successful application of modeling and simulation to complement clinical lactation data for enhanced knowledge of infant drug exposure are presented.

## Background

Since the 1990s, especially after the Women’s Health initiative (WHI) was established in 1992, there has been increased emphasis on generation of evidence to guide clinical practice and develop therapeutics for conditions that predominantly affect women^1^. Although this was ground-breaking, the WHI by design focussed on preventive strategies for chronic disorders like cancer, healthier ageing, and postmenopausal disorders, and thereby neglected to focus on the disposition and safety of drugs in Women of Child-Bearing Potential (WOCBP). Within the demographic of WOCBP, different categories of women exist; some who wish to conceive, and others who may be in different stages of pregnancy, postpartum and/or breastfeeding. It is estimated that more than half of all breastfeeding women worldwide require drug therapy^2^. However, in most situations, clear evidence quantitatively describing drug transfer from mother to breastfed infants (through breastmilk) and informing the clinical risk-to-benefit ratio does not exist. This evidence gap may preclude prescribing such therapeutics in the real-world setting. This applies to both established and newly approved therapeutics. A 2016 review by the FDA scrutinising the eligibility criteria of trials of 38 drugs approved between 2014 and 2017 observed that lactation was among the most frequent exclusion criteria^3^.

The main reasons for such exclusion largely relate to concerns about clinical trials in pregnancy and the resultant fetal drug exposure^4^. Similarly, ethical issues around inclusion of breastfeeding females are linked to the potential safety risk to the infant without providing any direct benefits. Infant exposure through breast milk is typically lower than that which occurs through placental transfer during pregnancy^5^, although important exceptions do exist. For example, bedaquiline concentrations were about 14-fold higher in breast milk relative to maternal plasma, in mothers treated for rifampicin-resistant tuberculosis; a breastfed infant whose mother was treated with this regimen reached similar plasma concentrations to the mother^6^. In clinical trials, expectedness is used to determine whether a reaction is an expected side effect of a drug or treatment. Case reports suggest that adverse drug reactions in breastfeeding infants are uncommon, but a clinical challenge is that non-specific symptoms (such as crying, irritability, etc) are relatively common in infants aged under 1 month; it can be difficult to determine whether any of these symptoms relate to drug exposure. Established pharmacovigilance systems enable reporting of potential transmammary adverse drug reactions, although rates of spontaneous reporting are low, and there is a bias towards more severe events such as respiratory depression^7^. Opioids and antidepressants or other central nervous system acting agents are most widely implicated, however there is paucity of knowledge on other drug classes, and it can be particularly challenging to investigate risk of subtle, long-term consequences^8^.

To date, relatively few clinical lactation studies are conducted during drug development and are generally performed as a post-marketing requirement or commitment (PMR or PMC), potentially because a sufficient amount of safety data at the therapeutically beneficial dose has been gathered by the time of approval or post-approval^9^. One review of approval letters of original New Drug Applications to the FDA between 2000 and 2022 report that only 18 included a lactation study as a PMR, 89% of which were requested after 2017^10^. Another review of US product labelling for 422 new molecular entities approved by the FDA between 2001 and 2020 found that only 23 (5%) included human lactation data^11^.

Systematic reviews of antiretrovirals^12^, drugs used to treat neglected tropical diseases, tuberculosis and malaria^13^, antihypertensives^14^ and antidepressants^15,16^ in lactating mothers report significant limitations in study design and quality. While there have been attempts to harmonise the approach to studying drugs in breastfeeding women since 2002^17,18^, there remains a clear need for key stakeholders to create a coordinated approach towards understanding the challenges involved and creating collaborative opportunities for future research in lactation. Furthermore, operational and methodological issues relating to the conduct of lactation studies remain an issue and require further consideration and discussion. Establishing clear consensus is timely given the recent US Food and Drug Administration (FDA) diversity action plan (DAP) draft guidance (July, 2024) that aims to improve enrolment of participants from underrepresented populations, including pregnancy and lactation, in clinical studies^19^.

To allow appropriate inclusion of breastfeeding women will require knowledge of drug disposition during lactation through pragmatic and feasible clinical trials. Guidance from the FDA released in 2005 and updated in 2019 recommends the conduct of lactation studies when *‘a drug under review for approval is expected to be used by women of reproductive age’*^20^.

Over the past few years, there have been an increasing number of publications making the case for the inclusion of pregnant and breastfeeding women in clinical trials^21–23^. Frequently, statements on ‘pregnancy and lactation’ group these together and give more emphasis to pregnancy, despite the fact that there are unique challenges to conducting trials in each WOCBP subpopulation. This article aims to redress the balance with a commentary on the current status of lactation trials and recommendations to improve inclusion.

## Perspectives are changing

**Fig. 1:**
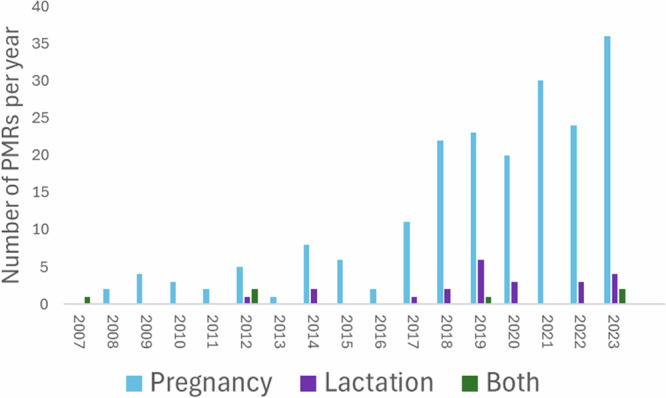
Number of FDA post marketing requirements (PMRs) registered over time focussing on pregnancy, lactation or both. This includes all PMRs, not only those exploring drug exposure. FDA Food and Drug Administration, PMRs post-marketing requirements.

The recent FDA DAP draft guidance (2024)^19^ and the International Council for Harmonisation of Technical Requirements for Pharmaceuticals for Human Use (ICH, a council comprised of global regulatory authorities and pharmaceutical industry leaders) E21: Inclusion of Pregnant and Breast-Feeding Individual in Clinical Trials final concept paper (2023)^24^ are both in effect a call to action for the enrolment of underrepresented populations, including demographics of age, sex, race, and ethnicity, in clinical studies during drug development. In many cases, pregnancy and lactation presents a continuum. Most lactating women will have recently been pregnant, and many pregnant women will wish to breastfeed. However, it is important to recognize that the two scenarios have important differences with respect to drug safety, pharmacokinetics and the ethics of including mother-infant pairs in clinical trials. Despite this, the number of clinical trials on lactation lag behind that of pregnancy-related ones. Figure 1 illustrates the total number of pregnancy and lactation specific PMRs related to new drug applications (NDAs) registered with the FDA from 2007 to July 2024^10^, extracted from the FDA’s accessible database^25^.

Furthermore, it needs to be recognized that many lactation studies are being conducted as exploratory studies by academic or clinical research groups, rather than investigational pragmatic trials led by industry. According to the clinical trial website (https://clinicaltrials.gov/) 18 lactation pharmacokinetic studies were conducted between 2010 and 2023. Currently, there are 24 ongoing studies with a focus on determining drug exposures in lactating mothers and/or breastfeeding infants.

This increased focus on lactation studies in more recent years is welcome, as these studies are needed to understand drug disposition in lactating WOCBP and their infants. For lactation, a key concern is significant secondary exposure in neonates and infants through breastfeeding following maternally administered drugs. In the case of anti-infectives, there is a risk of exposure to concentrations sufficient to risk selection for drug resistance should the infant acquire the infection being treated in the mother^26^. The “dose” consumed through breastfeeding is likely to be much lower than the oral dose proposed for drugs administered to neonates (birth to 28 days) and infants (28 days to 23 months), although non dose-dependent adverse drug reactions could still potentially occur, and in the case of anti-infectives, the below-target concentration in the breastfed infant may be sufficient to select for resistant organisms^27^. In May 2023, the FDA paediatric drug development draft guidance indicated that at least one clinical investigation in *“paediatric age groups (including neonates in appropriate cases) in which a drug is anticipated to be used”* should be conducted^28^. Industry is required to submit an initial paediatric study plan (iPSP) during early clinical development to ensure that paediatric subjects are considered early in the program. These requirements have also been adopted by the recent ICH E11a guideline^29^. The use of modelling and simulation to support dose recommendations in paediatrics is advocated^28,29^. Frequently, lactation studies focus on maternal pharmacokinetics and breastmilk transfer. However, the breastfed infant remains neglected relative to infants requiring direct treatment with therapeutics. Understanding the quantitative fraction of the dose transferred through breast milk to infants to assess any potential risks or benefits to them as a result of this secondary exposure is important to evaluate ideally during drug development or late phase studies.

## Exposure and safety data on drug use in lactation

Development and evaluation of a new drug requires understanding of its safety profile at a dose that can achieve therapeutic concentrations. This is particularly true for small molecule *versus* biologics, as studies to date suggest that very low levels of biologics are secreted into breastmilk, likely due to their large molecular weight and enzymatic degradation in the gastro-intestinal tract^30^. Typically, during drug development, safety data that inform the use of medicines during breastfeeding fall into three categories, including animal experiments, predictive computational tools that estimate the partitioning of a drug into the breast milk based on its physicochemical properties, and clinical exposure and safety data. In vitro, in vivo and in silico methods to study the transfer of maternal medication into human breast milk are available and have been described in detail in a recent review^31^. Although several in vitro models (animal and human mammary epithelial cell lines) are currently used, cell culture characterization, including transporter-mediated uptake across the in vitro blood-milk barrier, remains limited.

Non-clinical postnatal development studies are designed to collect initial information on exposure via milk in nursing pups, including evaluations of survival, growth and behaviour. Although animal data are used to assess whether a drug is likely to partition in human milk, they are not generally considered to be useful for extrapolating to drug concentrations in human milk. Indeed, the FDA’s Pregnancy and Lactation Labelling Rule (PLLR) recommends that in prescribing information *“Animal data not be included if human data exists”* and *“Animal data, when included, should only state presence or absence of drug in milk.”*^32^

Models to predict the milk-to-plasma concentration ratio (M:P ratio) of a drug based on the physicochemical characteristics of the drug (e.g., ionization, molecular weight, lipophilicity and protein binding affinity) are available and undergoing assessment^33,34^. Although these models have met with some success (depending on the characteristics of the drug), they are continuously evolving to accommodate the changing drug space (more metabolically stable drugs susceptible to transporter uptake and efflux)^35^. Currently, they are not considered sufficiently robust for prospective prediction of drug concentration profiles in human milk, especially if transporter-mediated secretion is involved. These predictive models can be integrated within a physiologically based pharmacokinetic (PBPK) modelling framework to simulate concentration-time profiles in mothers and breastfeeding infants^36,37^. PBPK models by their very nature are primed for this as they can account for the time-variant complex interplay between physiological parameters and drug-related characteristics in nursing mothers and infants.

## Regulatory perspectives on the conduct of clinical lactation studies

**Fig. 2:**
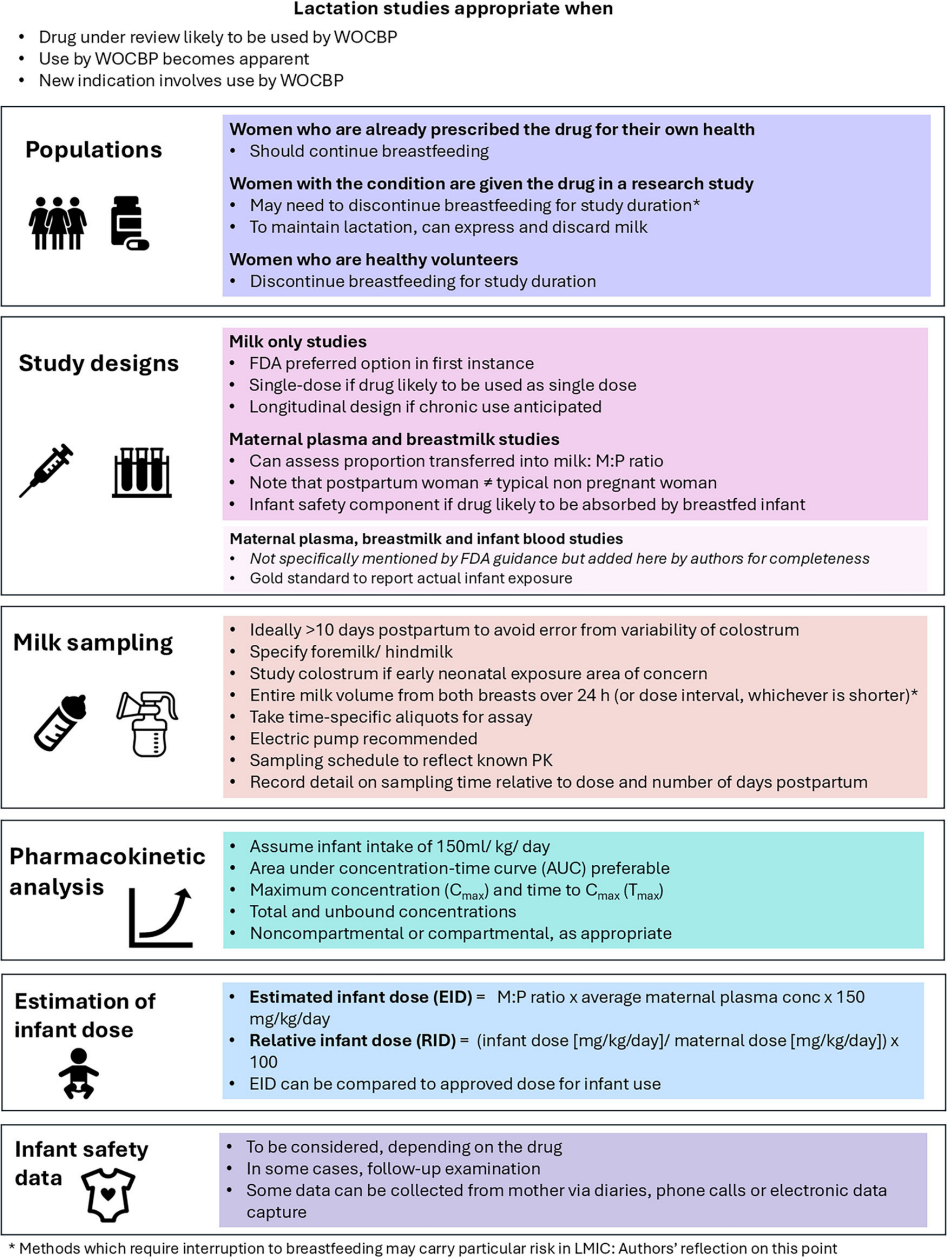
Summary of FDA 2019 draft guidance for lactation studies, with some additional author reflections. *C*_max_ maximum observed concentration, EID estimated infant dose, FDA Food and Drug Administration, M:P milk:plasma, PK pharmacokinetic(s), RID relative infant dose, *T*_max_ time to maximum observed concentration, WOCP women of childbearing potential.

Challenges associated with the conduct of clinical lactation studies to collect clinical exposure and safety data relate to ethical considerations and operational, enrolment and study design issues. These lactation studies may be recommended prior to approval when it is anticipated that a drug is likely to be used in WOCBP, or after approval when it becomes apparent that the drug is being used by these women, either for the original or a new indication. The FDA recommendations of 2019, including the commonly reported variables of milk to plasma ratio (M:P ratio), estimated infant dose (EID) and relative infant dose (RID) are summarised in Fig. 2.

In order to fully assess the potential of a drug to clinically affect a breastfeeding infant, the concentration-time profiles of the drug in breast milk and maternal plasma are required, with infant plasma where possible. Ideally, sufficient drug concentrations are measured to allow calculation of an area-under-the- concentration–time curve (AUC) for milk (AUC_milk_) and an average milk concentration (AUC/milk sampling duration), which can then be used to estimate a daily EID assuming a daily milk intake of 150 mL/kg^38^. Thereafter, the RID (the percent of the weight-adjusted maternal dosage consumed in breast milk over 24 h) is determined. A World Health Organization (WHO) Working Group proposed that drugs with an RID > 10% may not be safe in infants^39^, and that those with an RID greater than 25% should be avoided in nursing mothers^40^. However, it is important to note that these are arbitrary thresholds lacking any pharmacological basis and nor do they provide any indication of clinical adverse events in the breastfeeding infants^38^.

As per the FDA guidance, typically, a milk-only study is recommended in lactating women, unless “there is a reason to conduct another type of lactation study” such as a milk and plasma study (concern for accumulation) or a mother/infant pair study (information on accumulation in milk is available)^20^. Baseline demographics specified in the FDA guidance (maternal weight, age, gestational age at delivery, stage of lactation, smoking, alcohol intake, concomitant drugs, ethnicity, race, and any existing comorbidities) should be collected early in clinical development to identify potential covariates for dose adjustments in longitudinal studies. Further recommendations on additional baseline demographics to be collected to facilitate more robust modelling analyses are provided by Dodeja et al.^41^. Recommendations were also made about the type of milk to be collected (foremilk versus hindmilk) and the milk sampling method to be used^20^. This information is typically used to evaluate the safety of a drug when used by breastfeeding mothers - supported by other relevant data including drug physicochemical properties, mechanism of drug entry into breast milk, data from nonclinical studies and infant factors - and to develop recommendations to minimise infant exposure. Examples of physicochemical properties of drugs commonly used during lactation and their impact on clinical lactation recommendations can be found in a recent review by Alshogran et al.^42^.

In 2019, the WHO and International Maternal and Paediatric Adolescent AIDS Clinical Trials Network (IMPAACT) undertook a consensus meeting to define best practices with specific reference to antiretroviral therapies (ART)^18^. The consensus was that recruitment of pregnant and lactating women into clinical trials must be encouraged, and that both should be eligible for all Phase III ART trials and some Phase IIb clinical trials unless there is a compelling reason for exclusion. In addition, they recommended that enrolment of pregnant and lactating women be considered during early phase clinical trials to inform treatment and dosing decisions rather than conducting a lactation study as a PMR^43^. One of the most notable recommendations from the WHO and IMPAACT network was the use of PBPK modelling to support any potential dose adjustments in these populations in Phase II/III studies and Population PK (Pop-PK) modelling to support the follow up of sparse sampling studies. This is consistent with a stepwise approach proposed by Fairlie and colleagues^44^, and the ethical framework for such inclusion discussed in depth by Weld^45^.

The ICH E21 final concept paper aims to provide a globally-accepted framework and best practices to enable inclusion and/or retention of pregnant and breast-feeding individuals in clinical trials^24^. To support this ICH initiative, an Expert Working Group (EWG) encompassing individuals from diverse backgrounds relevant to the field was established to harmonise the relevant strategies and methodologies. One of the key deliverables of the EWG was to enable the safe conduct and robust data collection from clinical trials in pregnant and breast-feeding women to allow regulatory acceptance for inclusion in the product prescribing information. The use of existing data sources, including toxicology data from animals, real world evidence in pregnancy or breast-feeding for drugs of the same pharmacological group and available PBPK models, was proposed.

Even when a clinical study has been conducted, often no specific guidance is introduced into the prescribing information with respect to dosing recommendations for breastfeeding mothers. Thus, there is likely to be an increasing reliance on the use of PBPK and Pop-PK models to inform or support data from clinical lactation studies. Indeed, there have been a significant number of publications demonstrating the application of these approaches in the lactation area^36,46,47^.

## Role of modelling and simulation to support lactation data

**Fig. 3:**
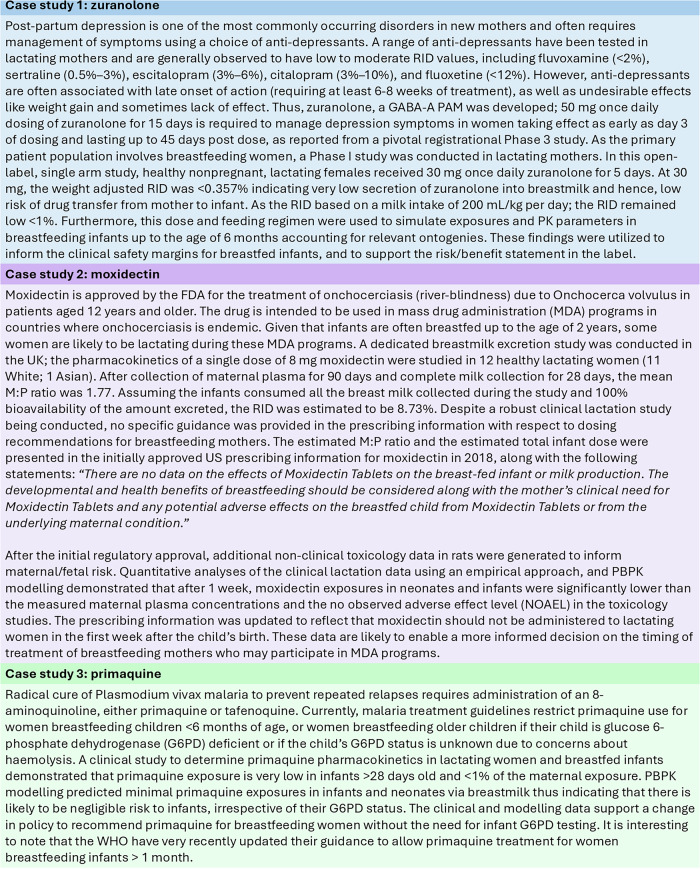
A Closer Look: Case Studies of Using Modelling and Simulation to Increase Clinical Knowledge of Drug Exposure in Lactating Women. References: Zuranolone^48,64,65^, Moxidectin^66,67^, Primaquine^46,68^. RID relative infant dose, GABA-A PAM Gamma-aminobutyric acid receptor positive allosteric modulator, Pop-PK population pharmacokinetics, FDA US Food and drug administration, MDA mass drug administration, M:P milk: plasma, NOAEL no observed adverse effect level, G6PD glucose 6-phosphate dehydrogenase, UK United Kingdom, US United States.

Pragmatic approaches such as modelling and simulation can be used to develop innovative clinical trial designs to enhance knowledge about drug exposure during lactation^48,49^. Specifically, PBPK models can be used to estimate and understand the transfer of drugs into breastmilk as well as identify drugs that may require clinical lactation studies. Both Pop-PK and PBPK can be used to predict exposures in breastfed infants, especially if a clinical lactation study includes mothers only or to support data generated from mother-infant pair studies. Herein, we describe three case studies where a combination of available data from investigational studies and verified Pop-PK or PBPK models have been used to support dosing recommendations for drug use in lactation in both drug development and global health settings. Figure 3 includes three different case studies that exemplify effective use of modelling and simulation tools to expand clinical knowledge of drugs used in lactating patients.

## Global regulatory perspectives on use of medicines during lactation

Whilst significant strides appear to have been made in countries with advanced economies as exemplified above, the regulatory landscape in Africa and other low-income countries, is still wanting. In 2017, WHO reported that although the majority of countries in Africa had National Medicines Regulatory Authorities (NMRA), most of them were not capable of performing the core functions expected of such a body due to limited resources^50^. Consequently, many low-income countries rely heavily on the decisions and approvals made by the more established regulatory bodies such as the FDA, the European Medicines Agency (EMA) and Japan’s Pharmaceutical and Medical Device Agency (PMDA).

Due to their limited capacity, it has been difficult for these NMRA in low-income countries to implement crucial regulations that would benefit their populations. Considering that breastfeeding is often the only affordable, feasible, acceptable, sustainable and safe (AFASS) feeding option for infants in these areas, these agencies should be supported and empowered to establish drug exposure and safety profile of drugs intended to be used by WOCBP. In turn this should encourage sponsors, including the pharmaceutical industry and academia to further invest in lactation studies during drug development stages, especially for therapeutics (and vaccines) that may be extensively prescribed in lactating women (antibiotics, antiretrovirals, antimalarials, analgesics, antihypertensives, antidepressants and others). This also becomes important during an era of global drug development and diversification of clinical studies by age and sex.

## Global networks - south to north learnings

Whilst it is imperative to reach a clear understanding of drug exposure to the breastfed infant in all parts of the world, the risks in advising a mother not to breastfeed are higher in regions where artificial feeding is not considered AFASS. This was shown in some of the early trials of antiretroviral therapy (ART) to prevent mother to child transmission of HIV, where artificial feeding was found to increase overall mortality rate^51,52^. The WHO has long recommended exclusive breastfeeding together with ART therapy to improve ‘HIV-free survival’ of the infant, in contrast to other regions where women living with HIV are recommended not to breastfeed because of the differences in the risk-benefit equation in those settings^53,54^. For this reason, many of the best examples of lactation studies have been undertaken on ART in Africa. Efforts to address the regional and global differences and maximize our learning, underpinned the establishment of the IMPAACT network in 2006 which included a global collaboration of investigators, institutions, and community representatives^55^. Other global networks include the ConcePTION Project (2019)^56^ and the US-based Maternal and Paediatric PRecision in Therapeutics (MPRINT) hub^57^.

**Fig. 4:**
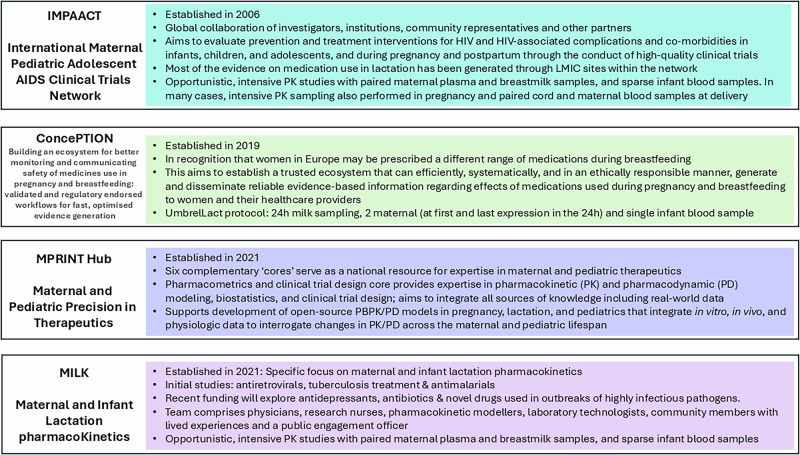
International networks focusing specifically on perinatal pharmacokinetics. IMPAACT International Maternal Paediatric Adolescent AIDS Clinical Trials Network, LMIC low and middle income country, MPRINT Maternal and Paediatric Precision in Therapeutics, MILK Maternal and Infant Lactation pharmacoKinetics. Figure References, IMPAACT^69^, CONCEPTION^70^, MPRINT^71^, MILK^60,61^.

These three large networks are led from North America and Europe, but their work has global relevance (Fig. 4). Furthermore, they focus on both pregnancy and lactation, often with a greater number of outputs relating to pregnancy. In contrast, the Maternal and Infant Lactation pharmacoKinetics (MILK) research programme specifically explores lactation pharmacology and is led from the Infectious Diseases Institute, Makerere University College of Health Sciences, Uganda. Studies have focussed on ART^58^, tuberculosis treatment^59^ and antimalarials^60^, and with recent funding will explore antidepressants, antibiotics and novel therapeutics used in outbreaks of highly infectious pathogens. The MILK team draws together physicians, research nurses, pharmacokinetic modellers, laboratory technologists, community members with lived experiences and a public engagement officer. This multi-disciplinary team has enabled model-based study design, and they have achieved full recruitment across all protocols^61^. In contrast to some of the ‘ethical and logistical’ challenges reported in other settings, their experience is that when provided with the right information, breastfeeding mothers are keen to participate in lactation research and to be part of the generation of the evidence which is currently lacking. Partnership with communities and a multifaceted communication strategy which includes mass media and social media have established credibility and trust to enable clear, ongoing dialogue with potential participants and their communities at all stages, from establishment of research priorities, protocol design and implementation and results dissemination^62^. In addition to a specific community advisory board, participatory approaches have enabled co-creation of key information, education and communication materials^63^, together with community empowerment. Low and middle income countries (LMIC) are often late to benefit from therapeutic advances, and initial evidence for novel treatments is often drawn from extremely different populations. Greater equity could be achieved if sponsors considered research in LMIC at an earlier stage, partnered with a commitment to make the drug affordable and accessible in these settings.

## Future perspective

Breastfeeding women will continue to form a key part of all societies in the world, and remain at risk of a wide range of medical conditions requiring medication use. Studies must be designed with the communities in mind, bring together diverse skill sets and be underpinned by strong stakeholder and community engagement. Furthermore, there is a rich opportunity for continued capacity strengthening and multi-directional knowledge exchange. Given the wealth of clinical experience with breastfeeding mothers requiring medication in Africa, South to North partnerships should enable transfer of the clinical skills required to deliver these studies to a high quality whilst minimising the potential burden on the mother and her child.

It has also been recognised by these networks that the use of modelling to inform or support clinical lactation studies in a drug development or clinical setting is essential. Thus, these collaborative efforts between clinicians, quantitative pharmacologists and modellers along with healthcare professionals in the field are likely to lead to an increasing application of these modelling approaches, drive decision making and support enrolment of this demographic in clinical trials during drug development. For this change to occur, regulators and policy makers *across the globe* need to become more familiar with modelling and simulation to gain confidence about its application in this area. Finally, to overcome the historical neglect of this population, the pharmaceutical industry and other funding agencies should continue to support lactation pharmacology research Table 1.

**Table 1:** Priorities for improving lactation pharmacokinetics research

Regulatory	• Support standardisation of methodologies for clinical lactation studies • Enhance the focus on conducting clinical lactation studies during drug development and as part of post-marketing requirements • National medicines regulatory agencies should advocate for the necessity for clinical lactation data prior to drug approval, attaining parity with the paediatric situation
Clinical study design	**•** Sampling from both mother and infant for a complete understanding of drug exposure profiles **•** Single or long-term use of the drug should guide the longitudinal time-frame of the study **•** Standardise breast milk sampling guidance. Define minimum common datasets necessary to support evidence-based guidance **•** Generate data to support standardisation – how much influence does timing of sample relative to feed really have? What is the impact of foremilk vs hindmilk for specific drugs?
Modelling and simulation	**•** Undertake model-informed plasma and breast milk sampling design **•** Use of population analysis and PBPK modelling to characterise drug transfer from plasma to breast milk, and the associated factors
Communication	**•** Provide clear, culturally sensitive informed consent process **•** Stakeholder engagement including community representatives, health and research agencies **•** Continued dialogue with stakeholders on integration of research findings into policy

## Data Availability

No datasets were generated or analysed during the current study.
